# A Knowledge-Enhanced Multimodal Framework with Genomic Reconstruction for DLBCL Drug Response Prediction

**DOI:** 10.34133/csbj.0191

**Published:** 2026-09-02

**Authors:** Xiaolu Xu, Yulong Li, Shuai Zheng, Chengjie Lu, Jingyi Zhou, Hongbin Lu, Beibei Zhu, Jiawen Yu, Zhaohong Geng

**Affiliations:** ^1^ School of Computer and Artificial Intelligence, Liaoning Normal University, Dalian 116029, China.; ^2^ Postdoctoral Workstation, Informedia Electronic Co., Ltd., Dalian 116001, China.; ^3^ Faculty of Medicine, Dalian University of Technology, Dalian 116024, China.; ^4^ Department of Hematology, The First Affiliated Hospital of Dalian Medical University, Dalian 116011, China.; ^5^ Department of Cardiology, The Second Affiliated Hospital of Dalian Medical University, Dalian 116023, China.

## Abstract

Diffuse large B-cell lymphoma (DLBCL) exhibits substantial biological heterogeneity, leading to pronounced variability in patient response to therapy. Accurate drug response prediction is therefore critical for precision treatment but remains challenging in clinical settings where genomic sequencing, a highly informative modality, is frequently incomplete. Existing methods, often developed from cell-line pharmacogenomic datasets or single-modality data, typically assume fully observed molecular profiles and thus show limited robustness under missing genomic data. To address this limitation, a knowledge-enhanced multimodal framework with genomic reconstruction (KeM-DRP) is proposed for individualized drug response prediction in DLBCL. The framework models the central role of genomics by integrating biological prior knowledge through a gene–pathway–biological process hierarchy, enabling robust representation learning from sparse observations. To compensate for missing genomic measurements, a cross-modal genomic compensation module reconstructs genomically informed latent features from routinely available clinical modalities. Furthermore, a genomics-guided adaptive fusion strategy dynamically integrates heterogeneous modalities conditioned on observed or reconstructed genomic representation. Experiments on a real-world DLBCL cohort demonstrate that KeM-DRP consistently outperforms competitive baselines. The reconstructed genomic representation represents most predictive utility, highlighting the robustness and practical value of the framework under incomplete genomic data.

## Introduction

Diffuse large B-cell lymphoma (DLBCL) is the most common and biologically heterogeneous subtype of non-Hodgkin lymphoma (NHL), accounting for approximately 30% to 40% of newly diagnosed NHL cases [1]. Although the R-CHOP regimen remains the standard first-line treatment, a substantial proportion of patients still experience primary resistance, relapse, or severe treatment-related toxicity [2]. These unfavorable outcomes highlight the need for accurate drug response prediction to support precision treatment in DLBCL [3].

Existing computational studies for drug response prediction have benefited from both large-scale public pharmacogenomic resources and patient-level clinical cohorts. While these data sources may overlap in practice and are increasingly integrated in recent studies, they differ in data scale, modality completeness, and proximity to routine clinical scenarios. Public-database-based approaches usually rely on large-scale pharmacogenomics resources, such as the Genomics of Drug Sensitivity in Cancer [4], the Cancer Cell Line Encyclopedia [5], and The Cancer Genome Atlas [6]. These datasets have enabled a wide range of machine learning and deep learning methods for drug response modeling. For example, Yang *et al.* [7] proposed NRL2DRP, which integrates gene mutations with protein interaction networks to learn functional context features for cell lines and then applies a support vector machine for prediction. Xu *et al.* [8] introduced an autoencoder-based framework with Boruta feature selection to identify informative genomic variables for downstream random forest prediction. Peng *et al.* [9] proposed NIHGCN, which models drug–cell-line interactions through heterogeneous graph convolution and neighborhood interaction modules. Cao *et al.* [10] developed BANDRP, a bilinear attention framework that integrates multi-omics features and molecular fingerprints for response prediction. Jiang *et al.* [11] presented DeepTTA, which combines Transformer-based drug structure encoding with transcriptomic profiles. He *et al.* [12] further proposed CODE-AE, a context-aware deconfounding autoencoder that attempts to transfer biological signals from cell line screening data to patient settings.

Despite the advantages of large-scale public datasets, translating models developed primarily from public pharmacogenomic resources to real-world DLBCL care remains nontrivial. Many existing methods are trained on cell-line screening data or data settings with relatively harmonized and complete molecular profiles, which may differ from routine clinical scenarios in terms of treatment context, modality availability, and missing-data patterns. Patient-level clinical cohorts therefore provide a complementary perspective by preserving treatment-specific response assessment and multimodal clinical evidence collected during real-world care, although they are also commonly constrained by limited sample size, retrospective design, and incomplete molecular profiling. Besides, these methods commonly assume complete molecular measurements, especially genomic profiles, whereas such data are often unavailable in routine clinical settings due to cost, tissue accessibility, and practical constraints. Given the critical role of genomic alterations in treatment response [13], this mismatch substantially restricts the clinical applicability of public-dataset-based models and highlights the need for multimodal approaches that can operate under incomplete data.

Clinical-data-based prediction methods typically leverage patient-derived information, such as genomic, pathological, immunological, and imaging features, to predict treatment responses. Gayvert *et al.* [14] developed a computational framework that combines monotherapy efficacy data and high-throughput screening with a random forest model to predict and validate synergistic drug combinations in BRAF-mutant melanoma. Kong *et al.* [15] proposed NetBio, a network-based machine learning framework that integrates protein interactome and transcriptomic data to identify immune-therapy-related gene modules for predicting patient responses to immune checkpoint inhibitors. Mo *et al.* [16] developed MOPCR, a subtype-specific multi-omics model that integrates features such as proliferation and methylation to predict pathological complete response in breast cancer neoadjuvant therapy. Lee *et al.* [17] developed a multimodal deep learning model that combines digital pathology and clinical data to predict immunochemotherapy response in DLBCL. Together, these studies demonstrate the clinical relevance of multimodal drug response prediction and highlight the value of integrating complementary patient-level evidence beyond single-modality modeling, particularly for clinically heterogeneous diseases such as DLBCL.

Nevertheless, applying multimodal drug response prediction to real-world DLBCL cohorts remains highly challenging. In routine practice, key modalities, particularly genomic sequencing, are frequently missing, while the number of eligible patients is often further reduced by strict inclusion criteria and follow-up requirements [18]. Consequently, prediction models must be developed in a small-sample setting with incomplete genomic information. Moreover, even when genomic data are available for a subset of patients, learning reliable representation from high-dimensional and sparse genomic features remains difficult. These challenges make it essential not only to exploit the information contained in routinely available modalities, but also to model genomic signals in a robust and biologically meaningful way.

Overall, existing multimodal strategies remain insufficient for this setting. Common approaches, such as direct feature concatenation or static feature aggregation, generally assume that all modalities are simultaneously available and fail to account for the central role of genomics in treatment response modeling. As a result, they are ill-suited to clinical cohorts with missing genomic data and offer limited capability for learning robust cross-modal interactions when critical modalities are absent [19–22]. These limitations motivate the development of a multimodal framework that can incorporate biological prior knowledge, compensate for missing genomic information, and adaptively integrate heterogeneous clinical modalities for robust drug response prediction.

Motivated by these challenges, we collected a real-world cohort of 798 patients with DLBCL from a large tertiary hospital in China, from which 125 eligible cases were included for analysis, and formulated the task as a multimodal learning problem under incomplete genomic data. Based on this setting, we propose KeM-DRP, a knowledge-enhanced multimodal framework with genomic reconstruction. KeM-DRP integrates biologically informed genomic representation learning, cross-modal genomic compensation, and genomics-guided multimodal fusion, enabling robust drug response prediction when genomic information is missing or only partially available. The major contributions of this work are summarized as follows:

• We establish a clinically motivated multimodal learning setting for DLBCL drug response prediction under incomplete genomic data and validate the proposed framework on a real-world cohort.
• A knowledge-enhanced genomic representation learning scheme is proposed to incorporate structured biological priors through a gene–pathway–biological process hierarchy, enabling more robust and biologically meaningful modeling of sparse genomic features.
• A cross-modal genomic compensation mechanism is developed to recover genomically related latent information from routinely available clinical modalities, thereby alleviating the impact of missing genomic measurements.
• A genomics-guided adaptive fusion mechanism is introduced to dynamically integrate heterogeneous modalities conditioned on observed or reconstructed genomic representations, leading to more robust drug response prediction under incomplete genomic information.

## Method

**Fig. 1.**
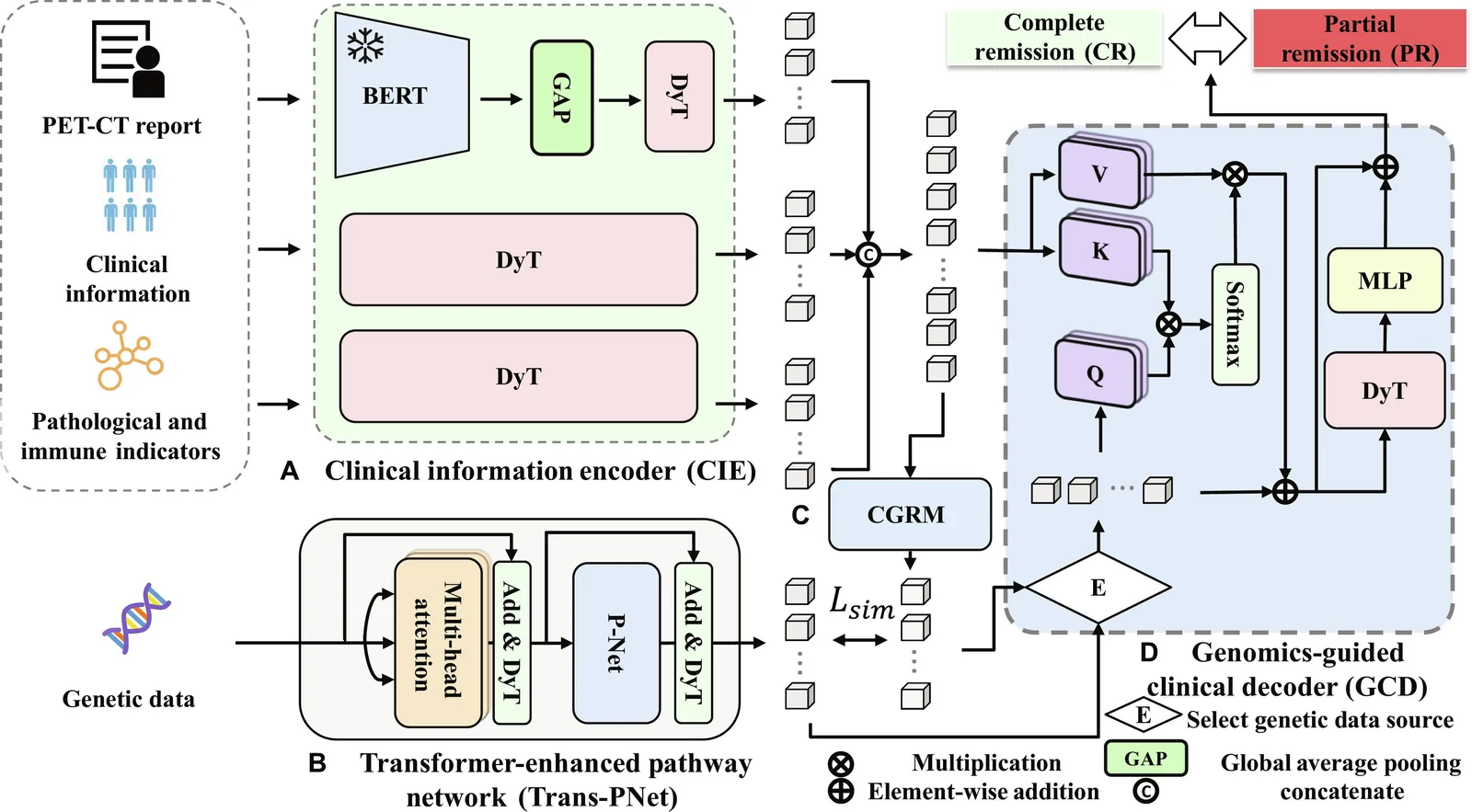
The overall structure of the KeM-DRP model, which mainly includes the following: (A) a clinical information encoder, (B) a Transformer-enhanced pathway network (Trans-PNet), (C) a clinico-genomic reconstruction module (CGRM), and (D) a genomics-guided clinical decoder (GCD).

As shown in Fig. 1, we propose KeM-DRP, a multimodal framework for precise drug response prediction in DLBCL by jointly modeling genomic sequencing and clinical multimodal data. To address the multifactorial nature of therapeutic response where patient cohorts are limited and sequencing data are frequently incomplete, the framework unifies knowledge-enhanced genomic representation learning, cross-modal genomic reconstruction, and adaptive multimodal fusion. Specifically, the Transformer-enhanced pathway network (Trans-PNet) performs biologically informed and pathway-aware genomic feature extraction from sequencing data, while a clinical multimodal encoder transforms heterogeneous patient-level evidence into structured latent representation. We further introduce a novel clinico-genomic reconstruction module (CGRM), which constructs pseudo-genomic features from clinical multimodal representation and aligns them with real sequencing-derived features through contrastive learning, thereby generating reliable substitutes for missing genomic information. Finally, a genomics-guided clinical decoder (GCD) adaptively integrates genomic and clinical representation to predict drug responses.

### Datasets and preprocessing

The dataset in this study was derived from a real-world cohort of 798 patients pathologically diagnosed with DLBCL who were admitted to the Department of Hematology of a large tertiary hospital in China between January 2017 and April 2025. From this initial cohort, 125 patients were included in the final analysis based on the following criteria: (a) confirmed diagnosis of DLBCL, (b) availability of treatment response evaluation based on positron emission tomography–computed tomography (PET-CT) before and after R-CHOP therapy, and (c) availability of key clinical modalities required for this study, including imaging reports and pathological/immunological indicators. Patients with incomplete follow-up data or missing essential clinical information were excluded. The study was approved by the institutional ethics committee and met the requirements of retrospective research. All patients received at least one cycle of R-CHOP and underwent PET-CT examinations both before and after treatment. Treatment response was evaluated by clinicians according to the Deauville 5-point scale of ^18^F-FDG PET/CT. The scale ranges from 1 to 5, with a score of 1 indicating no uptake and a score of 5 indicating markedly higher uptake than the liver or the appearance of new lesions. Scores of 1 to 3 were categorized as complete response (CR), while scores of 4 to 5 were classified as partial response (PR) [17]. This cohort reflects a clinically constrained patient-level setting, characterized by PET-CT-based response assessment after R-CHOP therapy and partially available genomic sequencing, which aligns with the practical motivation of KeM-DRP.

Table 1 presents the distribution of the clinical dataset, stratified by the availability of genomic sequencing information. Of the 125 enrolled patients, 86 (68.8%) achieved CR, while 39 (31.2%) reached PR. Within each response category, patients were further grouped based on whether sequencing data were available. Specifically, sequencing data were available for 37 CR patients (43.0%) and 22 PR patients (56.4%), while the remaining 49 CR patients (56.9%) and 17 PR patients (43.5%) lacked these data.

**Table 1.** Summary of dataset characteristics, including class distribution, gene sequencing status, and demographic information

Class	Gene sequencing	Count	Percentage	Age	Male	Class total
CR	✓	37	43.0%	63.64 ± 12.86	61.6%	86 (68.8%)
	×	49	56.9%			
PR	✓	22	56.4%	67.90 ± 9.85	56.4%	39 (31.2%)
	×	17	43.5%			
Total		125				

To evaluate model performance, stratified 5-fold cross-validation (5-CV) was employed. The dataset was partitioned into 5 folds while preserving the class distribution shown in Table 1. In each iteration, 4 folds were used for training and the remaining fold was used for testing, and the results were averaged over all 5 folds.

This study focused on 326 hot DLBCL genes. The full list of genes is provided in Table S1. To measure mutation burden, each mutation *i* was assigned a weighted deleterious score $M_{i}=F_{i}\times P_{i}$, where $F_{i}$ represents the observed mutation frequency and $P_{i}$ is the pathogenic probability predicted by MutationTaster [23]. Mutations predicted as “disease-causing” were considered potentially harmful. For genes with multiple mutations in a single patient, the scores were summed to obtain the cumulative mutation intensity (CMI) at the gene level:

$${\mathrm{CMI}}_{g}=\sum \left(M_{i}\right)=\sum \left(F_{i}\times P_{i}\right).$$ (1)

CMI, treated as a continuous variable, offers a refined quantification of the overall impact of gene mutations on patients.

The multimodal clinical data included patients’ imaging (PET-CT) reports at the pretreatment diagnostic stage, pathological and immunological indicators, and basic clinical information. The PET-CT reports detailed specific metrics, including the local SUVmax of lesions, the overall SUVmax, the sites of lymphoma involvement, and the size and number of lesions. The pathological and immunological indicators encompassed a comprehensive panel of immunohistochemical markers, comprising B-cell markers (CD19, CD20, and PAX5), germinal center markers (CD10 and BCL-6), an activated B-cell marker (MUM-1), a proliferation marker (Ki-67), apoptosis-related proteins (BCL-2, c-MYC, and P53), T/NK-cell markers (CD3, CD4, CD8, and CD56), as well as key molecules such as EBER. Moreover, flow cytometry data provided information on lymphocyte subpopulation distributions, including absolute counts and proportions of CD3^+^ T cells, CD4^+^/CD8^+^ T cell subsets, CD19^+^ B cells, and NK cells, as well as cytokine levels (IL-10, IL-1β, IL-6, and IL-8). All indicators from pathological and flow cytometry data were categorized into 3 levels: high, normal, and low. Basic clinical information included age, sex, and the presence of extranodal secondary involvement.

### Problem description

The goal of this study is to predict the drug response of DLBCL patients based on their genetic characteristics and clinical multimodal information. The gene matrix is denoted as $X_{\text{gene}}={\left[x_{1},\;\;x_{2},\;\;\ldots ,\;\;x_{i},\;\;\ldots ,\;\;x_{N}\right]}^{T}\in {\mathbb{R}}^{N\times d_{g}}$, which contains the $d_{g}$-dimensional gene features for *N* samples, where $x_{i}\in {\mathbb{R}}^{d_{g}}$ represents the gene feature vector for the *i*th sample. The clinical multimodal data include imaging reports $X_{\mathrm{PET}-\mathrm{CT}}=$$\left[r_{1},\;\;r_{2},\;\;\ldots ,\;\;r_{i},\;\;\ldots ,\;\;r_{N}\right]$, pathological and immunological indicators $X_{\mathrm{pai}}\in {\mathbb{R}}^{N\times d_{t}}$, and clinical information $X_{\mathrm{pat}}\in {\mathbb{R}}^{N\times d_{p}}$, where $r_{i}$ is the imaging report for the *i*th sample, $d_{t}$ is the dimension of pathological and immunological features, and $d_{p}$ is the dimension of clinical information. The corresponding drug response labels are defined as $Y={\left[y_{1},\;\;y_{2},\;\;\ldots ,\;\;y_{i},\;\;\ldots ,\;\;y_{N}\right]}^{T}\in {\left\{0,\;\;1\right\}}^{N}$, where $y_{i}$ denotes the binary label for the *i*th sample (0 for PR, and 1 for CR).

In real-world clinical settings, some modalities, particularly genomic data, may be partially missing. Let $M_{i}\subseteq$$\left\{\text{gene},\;\;\mathrm{PET}-\mathrm{CT},\;\;\mathrm{pai},\;\;\mathrm{pat}\right\}$ denote the set of available modalities for the *i*th sample. Our objective is to learn a predictive function $f\left(\cdot \right)$ that maps the available multimodal inputs to the drug response outcome:

$${\hat{y}}_{i}=f\left(\left\{x_{i}^{\left(m\right)}|\;\;m\in M_{i}\right\}\right)$$ (2)

such that the model can produce accurate and robust predictions under incomplete multimodal observations. The model parameters are learned by minimizing a loss function $L\left(y_{i},\;\;{\hat{y}}_{i}\right)$ over all samples.

### Transformer-enhanced pathway network

**Fig. 2.**
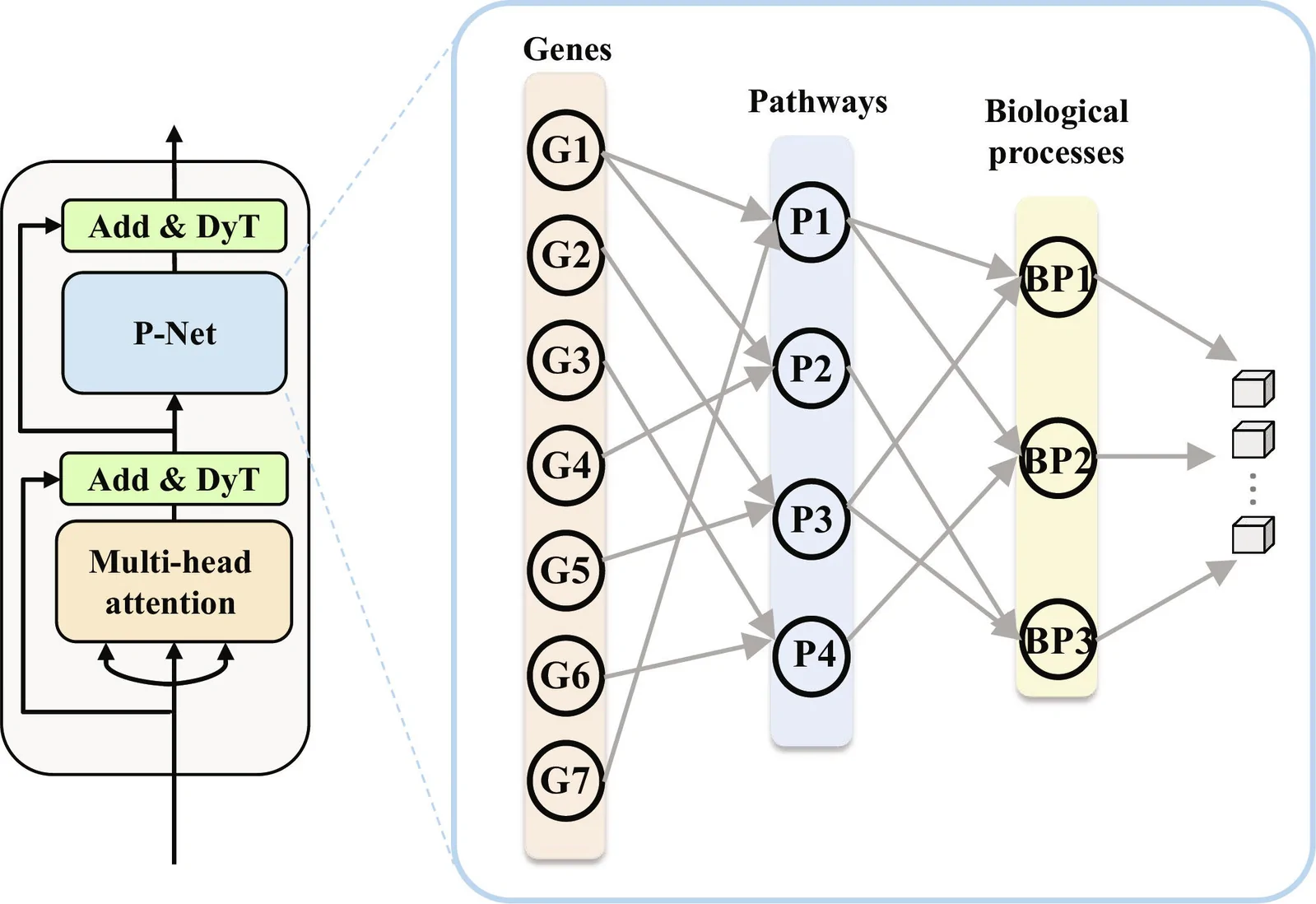
Transformer-enhanced pathway network (Trans-PNet).

Genomic information not only helps elucidate the molecular mechanisms underlying cancer but also complements conventional clinical modalities by providing deeper biological insights, thereby playing a critical role in drug response prediction. Recent studies have shown that interactions among genes, pathways, and biological processes substantially influence drug responses [24–26]. However, most existing methods fail to explicitly model such structured biological relationships, limiting the biological relevance and robustness of learned genomic representations. To address this limitation, we propose Trans-PNet (Fig. 2), a Transformer-enhanced pathway network that integrates self-attention with pathway-level priors organized in a gene–pathway–biological process hierarchy, enabling knowledge-enhanced genomic representation learning for more robust genomic feature modeling. The self-attention module captures potential interactions among genes by allowing gene representations to incorporate information from other genes, while the predefined gene–pathway–biological process topology in P-Net provides an additional biological constraint for organizing and propagating these learned representations. Therefore, the integration of self-attention and P-Net enables Trans-PNet to capture data-driven gene interactions while maintaining biologically informed structural guidance. This design constrains high-dimensional genomic representation learning within biologically meaningful subspaces, thereby improving stability and robustness.

Since genomic data are represented as high-dimensional feature vectors rather than natural language sequences, the input features are first serialized before being processed by the Transformer-based encoder. Given the serialized gene feature $G\in {\mathbb{R}}^{N\times d_{g}\times l}$,where *l* denotes the length of the gene feature sequence, gene–gene interactions are modeled via a self-attention mechanism:

$$F_{\mathrm{Att}}=\text{softmax}\left(\frac{\left({\mathrm{GW}}_{Q}\right){\left({\mathrm{GW}}_{K}\right)}^{⊤}}{\sqrt{d_{k}}}\right){\mathrm{GW}}_{V}.$$ (3)

where $W_{Q},W_{K},W_{V}\in {\mathbb{R}}^{l\times d_{k}}$ are projection matrices, which project the features into $d_{k}$-dimensional space to enhance gene-level representation capability.

To better integrate the original genomic features, a linear projection layer is applied to the attention output to align feature dimensions:

$$H_{\mathrm{Att}}=\text{Reshape}\left(\text{Linear}\left(F_{\mathrm{Att}}\right)\right),$$ (4)

where $F_{\mathrm{Att}}\in {\mathbb{R}}^{N\times d_{k}}$ and $H_{\mathrm{Att}}\in {\mathbb{R}}^{N\times d_{g}}$. The $\text{Reshape}\left(\cdot \right)$ operation reorganizes the projected features into gene-level representations, ensuring compatibility with the input G for element-wise residual addition.

In order to adaptively emphasize important gene interactions while preserving the original feature information, the attention output is added element-wise to the original input G through a residual connection and then normalized using DyT [27]:

$$G_{\text{enhanced}}=\mathrm{DyT}\left(H_{\mathrm{Att}}+G\right),$$ (5)

where $G_{\text{enhanced}}\in {\mathbb{R}}^{N\times d_{g}}$.

Although the self-attention mechanism can capture interactions among genes, it does not explicitly encode known biological structures. In contrast, prior biological knowledge, such as pathways and biological processes, provides valuable constraints for knowledge-enhanced genomic representation learning. Therefore, we incorporate the biologically informed sparse deep neural network PNet [28] to model the gene–pathway–biological process structure. Accordingly, the output of Trans-PNet is formulated as

$$F_{\text{gene}}=\mathrm{DyT}\;\left(\text{PNet}\left(G_{\text{enhanced}}\right)+G_{\text{enhanced}}\right).$$ (6)

Here, the residual connection is used to preserve the original gene representation while adding biologically structured residual information, thereby improving training stability and reducing information loss under limited data conditions.

In this way, Trans-PNet propagates and aggregates information among genes, pathways, and biological processes under biologically informed structural constraints. Specifically, pathways and biological processes are incorporated as structured priors that guide gene interactions, rather than being treated as independent input features. The resulting representation $F_{\text{gene}}$ integrates both self-attention-captured gene dependencies and explicit biological relationships, thereby improving biological relevance, representation stability, and robustness to overfitting.

### Clinical information encoder

In the context of clinical decision-making, physicians are required to integrate various heterogeneous information, such as imaging reports, pathology, and immunological indicators, to formulate a comprehensive diagnosis. However, traditional methods struggle to achieve effective cross-modal alignment and information integration, limiting the generalizability of predictive models. To overcome this challenge, we developed a clinical information encoder that maps multisource heterogeneous clinical data to an aligned feature space.

Specifically, the imaging report $X_{\mathrm{PET}-\mathrm{CT}}$ is encoded using a pretrained BERT model [29]:

$$H=\text{BERT}\left(X_{\mathrm{PET}-\mathrm{CT}}\right),$$ (7)

where $H\in {\mathbb{R}}^{N\times L\times d}$ denotes the token-level embedding sequence, with *L* being the sequence length and *d* being the hidden dimension. To facilitate subsequent cross-modal fusion and enhance feature robustness, we perform global average pooling on H along the sequence dimension to obtain a compressed feature vector:

$$H_{\mathrm{PET}-\mathrm{CT}}=\frac{1}{L}\sum_{j=1}^{L}H\left[:,\;\;j,\;\;:\right].$$ (8)

where $H_{\mathrm{PET}-\mathrm{CT}}\in {\mathbb{R}}^{N\times d}$ is the feature representation of the imaging report.

The features from all modalities, including the imaging report features $H_{\mathrm{PET}-\mathrm{CT}}$, pathology and immunological indicators $X_{\mathrm{pai}}$, and patient demographic information $X_{\mathrm{pat}}$, are then fed into the dynamic normalization module (DyT). This module maps multimodal clinical heterogeneous data into a unified feature space:

$$F_{\mathrm{PET}-\mathrm{CT}}={\mathrm{DyT}}_{\mathrm{PET}-\mathrm{CT}}\left(H_{\mathrm{PET}-\mathrm{CT}}\right),$$ (9)

$$F_{\mathrm{pai}}={\mathrm{DyT}}_{\mathrm{pai}}\left(X_{\mathrm{pai}}\right),$$ (10)

$$F_{\mathrm{pat}}={\mathrm{DyT}}_{\mathrm{pat}}\left(X_{\mathrm{pat}}\right).$$ (11)

Each DyT_*_ has independent parameters to adapt to the unique distribution characteristics of the corresponding modality. Moreover, the modules share a unified architectural design, mapping features from different modalities to a shared semantic space. Consequently, features $\left\{F_{\mathrm{PET}-\mathrm{CT}},\;\;F_{\mathrm{pai}},\;\;F_{\mathrm{pat}}\right\}$ are aligned in scale and semantics, providing a solid foundation for subsequent cross-modal fusion.

### Clinico-genomic reconstruction module

Genomic sequencing plays a critical role in cancer precision medicine, particularly in predicting patient responses to drug treatment [30]. Through in-depth analysis of genomic information, it is possible to identify molecular determinants related to drug metabolism, target interaction, and adverse reactions, thereby providing an important basis for personalized therapy. However, in real-world clinical settings, genomic sequencing is often unavailable for a substantial proportion of patients, which severely limits the applicability of genome-driven predictive models and further exacerbates the small-cohort challenge.

Motivated by recent advances in cross-modal representation learning and reconstruction [31–33], we design a CGRM to construct pseudo-genomic representation from imaging reports, pathological/immunological indicators, and clinical information. Specifically, CGRM is implemented as a multilayer perceptron (MLP) that maps the heterogeneous nongenomic features $F_{\mathrm{PET}-\mathrm{CT}}\in {\mathbb{R}}^{N\times L}$, $F_{\mathrm{pai}}\in {\mathbb{R}}^{N\times d_{t}}$, and $F_{\mathrm{pat}}\in {\mathbb{R}}^{N\times d_{p}}$ into a pseudo-genomic latent representation:

$$F_{\text{gene}}^{\prime }=\;\text{CGRM}\;\left(\text{Concat}\;\left(\left\{F_{\mathrm{PET}-\mathrm{CT}},\;\;F_{\mathrm{pai}},\;\;F_{\mathrm{pat}}\right\}\right)\right)$$ (12)

where $F_{\text{gene}}^{\prime }\in {\mathbb{R}}^{d}$ denotes the generated *d*-dimensional pseudo-genomic representation, with the same dimensionality as the genomic latent representation extracted by Trans-PNet in the “Transformer-enhanced pathway network” section.

Rather than recovering raw genomic measurements, this mapping aims to capture the latent clinico-genomic associations embedded in heterogeneous nongenomic modalities. By constructing pseudo-genomic representation and aligning them with real genomic latent representation during training, CGRM enables the model to exploit biologically relevant information even when sequencing data are missing. In this way, the module alleviates the dual bottleneck of limited cohort size and incomplete genomic profiling, increases effective data utilization, and improves the practicality and robustness of the framework in low-resource clinical settings.

### Genomics-guided clinical decoder

We further develop a GCD to selectively integrate heterogeneous clinical modalities under genomic guidance while adaptively handling incomplete sequencing data. Instead of directly concatenating multimodal features, GCD employs a cross-attention-based dynamic weighting mechanism to modulate modality contributions and suppress irrelevant noise. In addition, a conditional selection strategy is introduced to dynamically choose between real genomic latent and pseudo-genomic representations according to the availability of sequencing data:

$$F_{\text{gene}}^{\text{used}}=\left\{\begin{array}{ll} F_{\text{gene}}, & \text{if}\;E=1\\ F_{\text{gene}}^{\prime }, & \text{if}\;E=0 \end{array}\right.,$$ (13)

where $E\in \left\{0,\;1\right\}$ is a binary indicator variable denoting the presence (*E* = 1) or absence (*E* = 0) of the real genomic sequencing data.

Specifically, the multimodal clinical feature is first constructed as

$$F_{\text{clinical}}=\text{Concat}\;\left(F_{\mathrm{PET}-\mathrm{CT}},\;\;F_{\mathrm{pai}},\;\;F_{\mathrm{pat}}\right).$$ (14)

To achieve deep interaction between multimodal clinical information and genomic features, a cross-attention mechanism is integrated for feature fusion. The cross-attention operation is as follows:

$$F_{\text{cross}}=\text{softmax}\left(\frac{\left(F_{\text{gene}}^{\text{used}}W_{Q}^{\prime }\right){\left(F_{\text{clinical}}W_{K}^{\prime }\right)}^{⊤}}{\sqrt{d_{k}}}\right)\left(F_{\text{clinical}}W_{V}^{\prime }\right).$$ (15)

where $W_{Q}^{\prime }\in {\mathbb{R}}^{d\times d_{k}}$, $W_{K}^{\prime },\text{and}\;W_{V}^{\prime }\in {\mathbb{R}}^{d_{c}\times d_{k}}$ denote learnable projection matrices that map the features into a $d_{k}$-dimensional space to enhance the representational ability. The genomic modality serves as the query to locate relevant information within the multimodal clinical features, which act as keys and values to provide rich contextual semantics. The model thereby adaptively extracts and fuses the most pertinent clinical information from the genomic perspective.

To align the representation with the genomic feature space, the cross-modal feature $F_{\text{cross}}$ is reshaped from ${\mathbb{R}}^{N\times L\times d}$ to ${\mathbb{R}}^{N\times d}$ via a pooling-based $\text{Reshape}\left(\cdot \right)$ operation, which aggregates token-level information into a fixed-length representation. To focus the fusion process more on the genomic modality, a residual connection mechanism is employed:

$$F_{\text{fused}}=\text{Reshape}\;\left(F_{\text{cross}}\right)+F_{\text{gene}}^{\text{used}}$$ (16)

This approach ensures that genomic information is preserved during the fusion, while also incorporating the semantic enhancements introduced by cross-modal interaction. Finally, the fused features are denoised through an MLP and a dynamic normalization function:

$$F_{\text{out}}=\mathrm{MLP}\;\left(\mathrm{DyT}\left(F_{\text{fused}}\right)+F_{\text{fused}}\right).$$ (17)

where $F_{\text{out}}$ represents the final probability used for drug response prediction.

### Hybrid contrastive binary loss

The overall training objective consists of 2 components: a binary cross-entropy loss for supervised prediction and a clinico-genomic alignment loss for representation-level consistency. The binary cross-entropy loss is defined as

$$\begin{array}{c} L_{\mathrm{BCE}}=-\frac{1}{N}\sum_{i=1}^{N}\left[y_{i}\text{log}{\hat{y}}_{i}+\left(1-y_{i}\right)\text{log}\left(1-{\hat{y}}_{i}\right)\right], \end{array}$$ (18)

where *N* denotes the total number of samples, and $y_{i}$ and ${\hat{y}}_{i}$ represent the ground-truth label and predicted probability of the *i*th sample, respectively.

To alleviate the adverse effect of incomplete sequencing data, we further introduce a contrastive alignment constraint at the feature level. During training, for samples with available genomic sequencing data, the pseudo-genomic representation constructed by CGRM is encouraged to align with the real genomic latent representation learned by Trans-PNet through cosine-based similarity supervision:

$$L_{\text{align}}=1-\frac{1}{M}\sum_{j=1}^{M}{\text{cos}}_{\mathrm{sim}}\;\left(F_{\text{gene},j},\;\;F_{\text{gene},j}^{\prime }\right),$$ (19)

where $F_{\text{gene},j}$ and $F_{\text{gene},j}^{\prime }$ denote the real genomic latent and the pseudo-genomic representations of the *j*th sample, respectively; *M* is the number of samples with genomic sequencing data; and $\cos_{\mathrm{sim}}\left(\cdot ,\;\cdot \right)$ denotes the cosine similarity function. This alignment loss narrows the representation gap between real and reconstructed genomic features, thereby enabling the model to exploit genomically relevant information even when sequencing data are missing.

Accordingly, the total loss is defined as

$$L_{\text{total}}=\left(1-\eta \right)L_{\mathrm{BCE}}+\eta L_{\text{align}},$$ (20)

where η is a trade-off coefficient that balances predictive supervision and clinico-genomic alignment. The effect of η is analyzed in the “Experimental Results” section.

### Model complexity

The model complexity can be summarized as

$$O\left(B\left(\mathrm{Hd}\;d_{\text{gene}}^{2}+\sum_{l}E_{l}+{\mathrm{MT}}^{2}D\right)\right),$$ (21)

where $d_{\text{gene}}$ denotes the dimensionality of gene features, $E_{l}$ represents the number of biologically defined nonzero connections in the P-net, and *T* is the token length of $X_{\mathrm{PET}-\mathrm{CT}}$.

The gene attention module and BERT encoder exhibit quadratic complexity [$O\left(d_{\text{gene}}^{2}\right)$ and $O\left(T^{2}\right)$, respectively], while the P-net backbone remains sparse with complexity $O\left(\sum_{l}E_{l}\right)$. Although the full model contains ∼102.56M parameters, only ∼0.29M are trainable since the BERT encoder is frozen. The P-net backbone itself contains only a few thousand parameters due to biologically constrained sparse connections. Overall, the model balances efficiency and expressiveness by combining sparse pathway structure with attention-based components. The detailed implementation is presented in Algorithm 1.


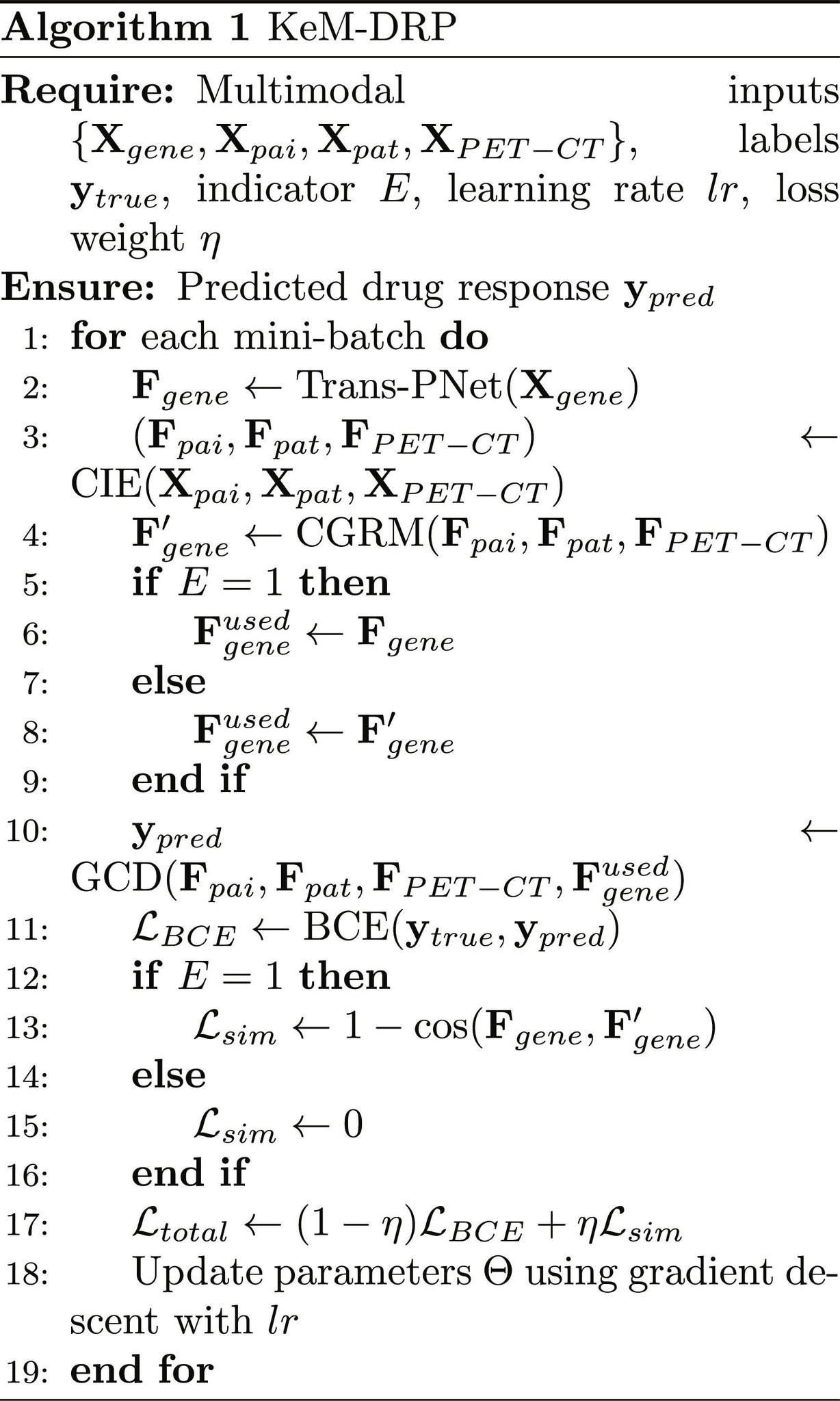


## Experimental Results

### Implementation details and evaluation metrics

#### Implementation details

This study constructs the model based on the TensorFlow framework and is conducted on an NVIDIA GeForce RTX 4070 Ti SUPER GPU. Cross-validation and random selection of 20 seeds are used for repeated experiments. The chosen optimizer is ADAM. The dataset is divided into training and testing sets in an 8:2 ratio. During the training phase, a total of 100 epochs are iterated. The learning rate follows a dynamic decay strategy, starting at 0.01 and gradually decreasing as the epoch increases. During model optimization, we adopted stratified sampling to preserve the original class distribution, ensuring that the model was trained on data with a consistent class composition.

#### Evaluation metrics

To comprehensively evaluate the drug response prediction performance, we employed 6 standard metrics, including accuracy, precision, recall, F1-score, the area under the receiver operating characteristic curve (AUC), and the area under the precision–recall curve (AUPR).

### Performance comparison

KeM-DRP was compared against 5 representative baseline models, including MCMoE [34], DeepTTC [11], BANDRP [10], SVM [7], and MLP. These baselines were selected based on their relevance to multimodal learning and drug response prediction tasks, as well as the availability of reproducible implementations, thereby ensuring fair, consistent, and comparable evaluations. For the conventional machine learning baselines, namely, SVM and MLP, heterogeneous multimodal features were integrated via feature-level fusion and represented as a unified input vector. In contrast, for DeepTTC and BANDRP, nongenomic modalities were incorporated into the drug branch in accordance with their architectural designs. All comparisons were conducted using 5-CV.

Consistent experimental results showed that KeM-DRP outperformed all baselines in terms of AUC, AUPR, accuracy, F1-score, and precision (Table 2), demonstrating its robust performance for DLBCL drug response prediction. Considering the moderately imbalanced CR/PR distribution, recall and precision were interpreted jointly rather than relying on a single metric. Notably, although DeepTTC, BANDRP, and MCMoE achieved higher recall values, this was accompanied by clear reductions in precision, AUC, and accuracy, suggesting less reliable classification boundaries. In contrast, KeM-DRP maintained a more balanced and stable performance profile across evaluation metrics.

**Table 2.** The performance on the DLBCL dataset via 5-fold cross-validation (%). Boldface is used to indicate the best-performing value for each evaluation metric.

Metric	SVM	MLP	DeepTTC	BANDRP	MCMoE	Ours
Accuracy	67.79 ± 7.95	64.15 ± 7.98	64.35 ± 3.29	66.92 ± 8.22	70.51 ± 8.22	**78.50** ± **5.19**
Precision	75.47 ± 6.25	71.54 ± 4.29	67.62 ± 0.79	71.47 ± 4.18	75.76 ± 7.13	**86.82** ± **8.35**
AUC	67.52 ± 8.76	63.11 ± 8.04	49.35 ± 15.96	59.50 ± 10.80	65.62 ± 14.9	**80.33** ± **8.75**
F1	76.92 ± 6.75	74.89 ± 6.50	77.98 ± 2.77	77.97 ± 5.75	79.77 ± 6.56	**83.67** ± **5.73**
AUPR	82.48 ± 7.17	80.59 ± 5.32	74.47 ± 10.64	73.95 ± 7.06	82.26 ± 7.64	**87.83** ± **6.65**
Recall	79.08 ± 9.98	78.92 ± 10.06	**92.42** ± **7.28**	86.92 ± 12.53	86.00 ± 12.69	82.92 ± 13.97

Quantitatively, KeM-DRP achieved absolute improvements of 12.81 percentage points in AUC over the strongest baseline SVM and 5.57 percentage points in AUPR over MCMoE, and yielded a 30.98 percentage points higher AUC than DeepTTC. These substantial margins indicate that KeM-DRP can more effectively exploit multimodal information under small-cohort conditions. We hypothesize that baseline methods such as BANDRP and DeepTTC underperformed partly because direct or insufficiently selective multimodal fusion may amplify irrelevant noise and dilute critical modality-specific signals. In contrast, KeM-DRP mitigates this limitation through pseudo-genomic construction and genomics-guided dynamic multimodal decoding. Collectively, these results suggest that the proposed framework is well-suited to DLBCL drug response prediction in limited-sample, heterogeneous multimodal settings.

### Stability experiments

To evaluate model stability, we repeated the entire training and evaluation process using 20 different random seeds, thereby assessing the robustness of KeM-DRP in clinically realistic application scenarios. As shown in Table 3, the proposed framework achieved the best performance on most evaluation metrics. Compared with the best-performing baseline (MCMoE), it showed higher accuracy and F1, with improvements of 8.85 and 6.34 percentage points, respectively, while showing slightly lower AUPR by 0.23 percentage points. In addition, lower standard deviations in AUC, F1, AUPR, and recall further indicated superior consistency and robustness across repeated runs.

**Table 3.** The stability results on the DLBCL dataset across 20 random seeds (%). Note: ↑ and ↓ indicate that the corresponding method is significantly higher or lower than Ours, respectively, according to a 2-sided paired *t* test. *, **, and *** denote *P* < 0.05, *P* < 0.01, and *P* < 0.001, respectively. Boldface is used to indicate the best-performing value for each evaluation metric.

Metric	SVM	MLP	DeepTTC	BANDRP	MCMoE	Ours
Accuracy	66.54 ± 8.19***^↓^	62.11 ± 9.77***^↓^	69.61 ± 4.82***^↓^	65.96 ± 8.94***^↓^	73.65 ± 6.27***^↓^	**82.50** ± **5.51**
Precision	74.57 ± 7.76***^↓^	70.31 ± 8.82***^↓^	70.70 ± 4.63***^↓^	72.80 ± 9.57***^↓^	81.81 ± 12.35	**85.20** ± **7.31**
AUC	64.83 ± 9.09***^↓^	58.78 ± 11.19***^↓^	51.61 ± 13.12***^↓^	62.95 ± 13.30***^↓^	78.55 ± 14.54	**82.43** ± **6.98**
F1	76.53 ± 6.28***^↓^	74.33 ± 7.25***^↓^	81.49 ± 3.38***^↓^	77.03 ± 7.12***^↓^	81.52 ± 5.12***^↓^	**87.86** ± **3.93**
AUPR	82.19 ± 7.86**^↓^	79.99 ± 8.22**^↓^	73.66 ± 8.97***^↓^	78.88 ± 9.74***^↓^	**88.69** ± **9.47**	88.46 ± 6.38
Recall	79.23 ± 8.30***^↓^	79.52 ± 8.48***^↓^	**96.78** ± 6.13*^↑^	83.64 ± 12.61*^↓^	85.61 ± 15.75	91.47 ± 6.63

The strong stability of KeM-DRP is related to its overall design, which combines biologically informed genomic modeling, cross-modal genomic compensation, and genomics-guided multimodal fusion. These components help improve robustness under limited samples and incomplete genomic data by enhancing representation quality and facilitating more effective use of heterogeneous clinical information. Overall, KeM-DRP not only achieved strong predictive performance in cross-validation but also demonstrated consistent stability across repeated independent experiments. These findings support the potential applicability of KeM-DRP for real-world DLBCL drug response prediction in clinical practice.

**Fig. 3.**
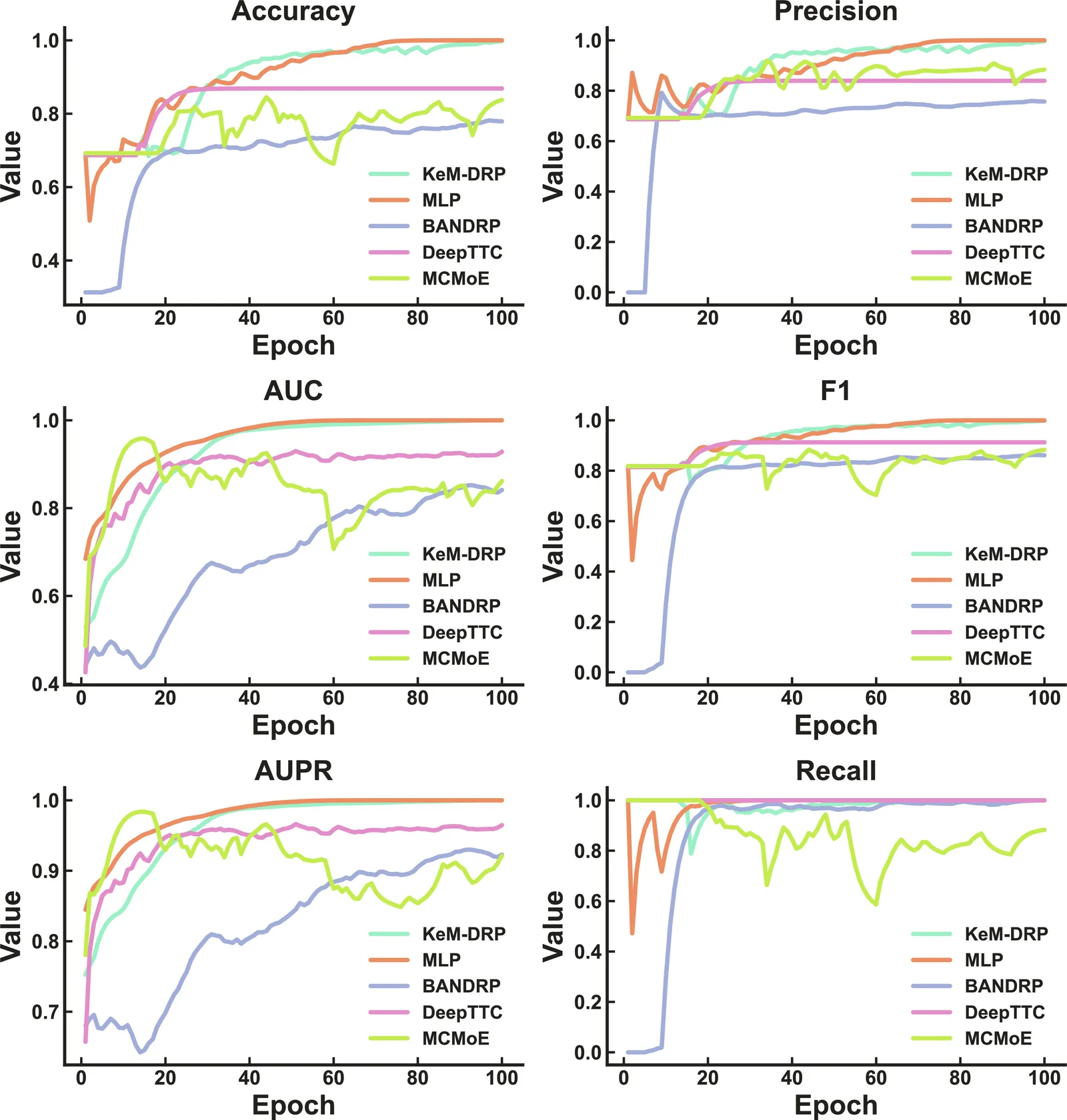
Performance trajectories of neural network-based models over epochs across 6 evaluation metrics.

Figure 3 shows the performance trajectories of 4 trainable models across epochs for one representative run out of the 20 repeated experiments, excluding SVM due to its noniterative optimization. While KeM-DRP achieves performance comparable to, or slightly below, MLP on some metrics during training, this observation is consistent with our design objective rather than a limitation. Combined with the results in Tables 2 and 3, these observations suggest that KeM-DRP tends to maintain more stable performance under incomplete multimodal inputs, rather than optimizing performance solely under fully observed settings. In contrast to models that rely more heavily on dominant modalities, KeM-DRP maintains stable predictive performance across varying modality availability, particularly when genomic data are partially missing.

**Fig. 4.**
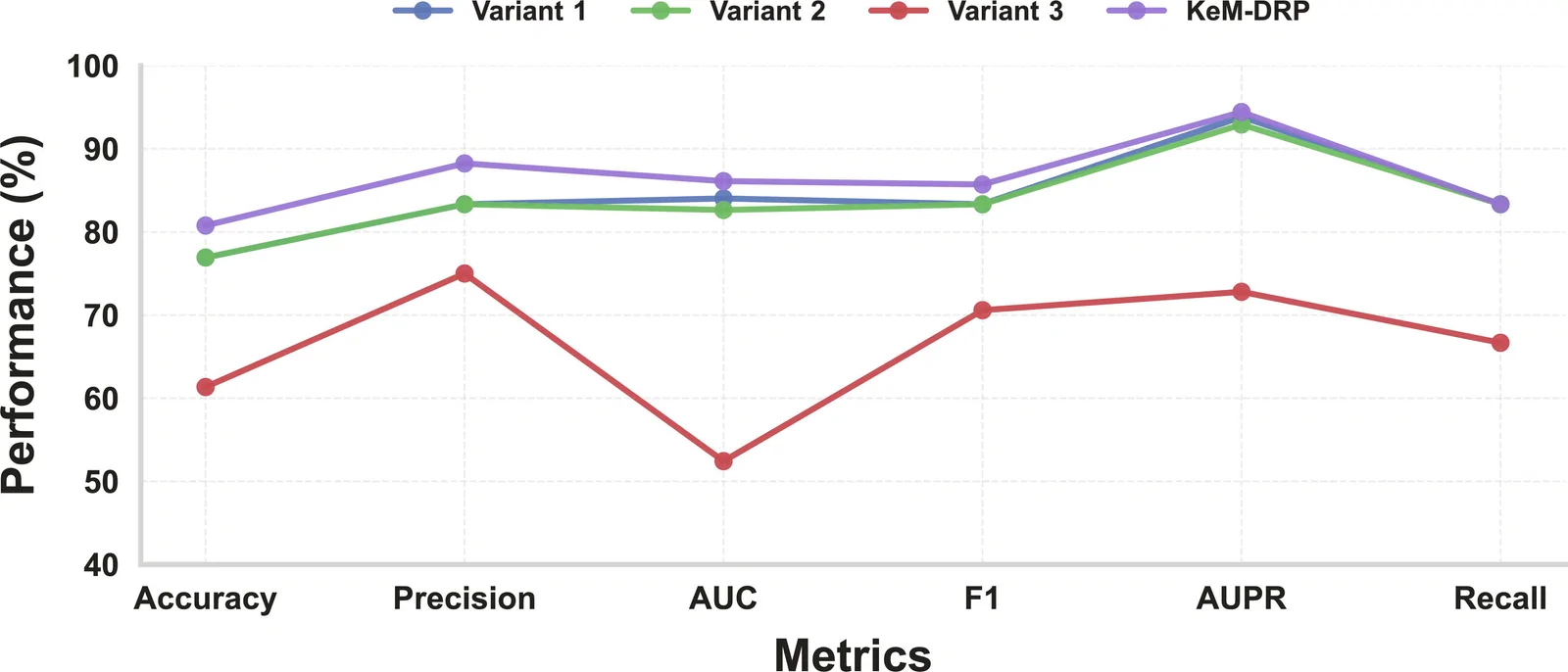
The results of the ablation experiments conducted to assess the contribution of each component in KeM-DRP.

To evaluate the contributions of the key components in KeM-DRP, we designed 3 ablation variants, as summarized in Table 4. Each variant was constructed by removing or replacing one major module while keeping the remaining architecture unchanged, thereby allowing us to assess the individual contribution of each component to the overall performance, based on a single train–test split, as illustrated in Fig. 4.

**Table 4.** Details of variant models in ablation studies

Model	Trans-PNet	GCD	DyT
Variant 1	×	✓	✓
Variant 2	✓	×	✓
Variant 3	✓	✓	×
KeM-DRP	✓	✓	✓

The ablation results show that all examined modules make substantial contributions to the final predictive performance. Variant 1 removed Trans-PNet and replaced the biologically constrained genomic encoder with a simplified alternative. The resulting performance decline indicates that pathway-guided genomic representation learning is essential for capturing informative gene-level dependencies and improving prediction accuracy. Variant 2 removed the GCD, resulting in notably worse performance than the full model. This observation highlights the critical role of genomics-guided cross-attention-based dynamic weighting in selectively integrating heterogeneous clinical modalities with genomic information while reducing irrelevant noise during multimodal fusion. Variant 3, which replaced DyT with conventional normalization, yielded the lowest performance. This outcome supports the importance of adaptive normalization for improving robustness when handling heterogeneous clinical inputs. Overall, the ablation results suggest that selective and genomics-aware multimodal interaction is crucial for robust prediction under heterogeneous clinical data conditions.

Notably, the CGRM was retained in all ablation settings. Because this module is tightly coupled with the incomplete-sequencing handling pipeline and functions as a foundational mechanism for pseudo-genomic representation construction, CGRM was not independently removed in the current study. Nevertheless, the contribution of this retained component is indirectly reflected in the overall robustness of the complete model under missing genomic data.

### Modality ablation study

To investigate the contribution of each modality and evaluate the robustness of the proposed framework under incomplete modality settings, we conduct a modality ablation study by removing one modality at a time, including gene, clinical information, pathological, and immunological data, and PET-CT reports, as summarized in Table 5. The full model using all modalities is included as the reference baseline.

**Table 5.** Performance comparison across different data modalities (%). Boldface is used to indicate the best-performing value for each evaluation metric.

Modality	Accuracy	Precision	AUC	F1-score	AUPR	Recall
Full model	**80.77**	**88.24**	**86.11**	**85.71**	**94.42**	83.33
Without gene	65.38	68.00	62.50	79.07	80.86	**94.44**
Without clinical information	61.54	75.00	56.60	70.59	76.70	66.67
Without pathological and immunological	73.08	78.95	61.81	81.08	77.54	83.33
Without PET-CT reports	69.23	81.25	78.13	76.47	87.80	72.22

In comparison, removing clinical information leads to the most substantial performance drop in accuracy and AUC, suggesting that clinical data offer crucial contextual support for prediction. Excluding pathological and immunological data results in a noticeable decline in AUC despite relatively high recall, indicating that this modality contributes to the model’s discriminative capability and complements the knowledge-enhanced genomic representation based on the gene–pathway–biological process hierarchy. Furthermore, removing PET-CT reports leads to a moderate performance change, with relatively high AUC but reduced recall, suggesting that imaging-derived semantic information provides additional but less dominant cues.

Overall, these results demonstrate that while all modalities contribute to predictive performance, the full multimodal setting achieves the most balanced and superior performance. The degradation observed after removing the gene modality underscores the importance of genomic information, while the performance drops caused by removing other modalities further confirm their complementary contributions to the proposed framework.

### Quality of reconstructed genomic features

To evaluate the quality of reconstructed genomic features, we conducted an experiment in which the original genes in the test set were replaced with reconstructed ones generated by our model. For each sample, we substituted the observed genes with their reconstructed counterparts while keeping all other features unchanged, and then evaluated model performance using the same metrics. In addition, the reconstructed genomic features achieved an average cosine similarity exceeding 0.9 with the original gene features, indicating high reconstruction fidelity.

As shown in Table 6, the reconstructed genomic features resulted in relative decreases of 4.76% in accuracy, 5.63% in precision, and 2.78% in F1-score compared with the original genes, while recall remained unchanged. In contrast, they achieved relative improvements of 3.24% in AUC and 1.47% in AUPR, suggesting that the reconstructed features provide a smoother and more robust representation for prediction. This trend may be explained by the fact that the proposed framework, where knowledge-enhanced genomic representation learning, guided by the gene–pathway–biological process hierarchy, promotes biologically consistent and smoothed representations that prioritize global discrimination over threshold-dependent metrics. Meanwhile, the cross-modal genomic compensation mechanism recovers missing genomic information from clinical data, capturing underlying genomic patterns despite imperfect reconstruction.

**Table 6.** Performance comparison between original genes and reconstructed genes (%). The up arrow (↑) indicates an increase relative to the corresponding baseline value, whereas the down arrow (↓) indicates a decrease relative to the corresponding baseline value.

Metric	Original genes	Reconstructed genes
Accuracy	80.77	76.92 ↓
Precision	88.24	83.33 ↓
Recall	83.33	83.33
F1-score	85.71	83.33 ↓
AUC	86.11	88.89 ↑
AUPR	94.42	95.81 ↑

In addition, we further evaluate the robustness of the proposed framework under different levels of genomic missingness by varying the missing ratio from 0% to 90%. As shown in Table 7, the model maintains relatively high performance even under larger genomic missing ratios, with the 0% missing ratio serving as the complete-genomic-data reference. Although performance fluctuates as the missing ratio increases, the model remains competitive even under high missing ratios, with consistently high recall. This result further demonstrates that the proposed framework can effectively leverage complementary information from other modalities to mitigate the impact of missing genomic data.

**Table 7.** Performance under different genomic missing ratios (%)

Missing Ratio	Accuracy	Precision	AUC	F1-score	AUPR	Recall
0%	80.77	88.24	86.11	85.71	94.42	83.33
10%	84.62	88.89	88.89	88.89	95.37	88.89
30%	76.92	87.50	79.17	82.35	87.93	77.78
50%	73.08	73.91	82.64	82.93	90.37	94.44
70%	80.77	78.26	77.08	87.80	83.70	100.00
90%	73.08	76.19	84.03	82.05	93.40	88.89

Overall, these results indicate that the reconstructed genomic features preserve most of the discriminative information required for downstream tasks, supporting their effectiveness as substitutes for real gene sequencing data in low-resource settings.

### Temporal generalization evaluation

To further evaluate the robustness of our model under realistic clinical settings, we conduct an additional experiment using a temporal split, where patients are partitioned based on their data collection time. Specifically, earlier cases are used for training, while later cases are reserved for testing, thereby simulating a prospective deployment scenario. The results are shown in Table 8.

**Table 8.** Performance under temporal split setting (%). Boldface is used to indicate the best-performing value for each evaluation metric.

Metric	SVM	MLP	DeepTTC	BANDRP	MCMoE	Ours
Accuracy	51.22	51.22	65.85	65.85	82.93	**82.93**
Precision	64.29	64.29	67.50	67.50	**92.00**	81.82
AUC	45.88	43.41	61.54	59.62	85.71	**89.70**
F1	64.29	64.29	79.41	79.41	86.79	**88.52**
AUPR	73.54	67.92	78.60	78.21	85.57	**92.67**
Recall	64.29	64.29	96.43	96.43	82.14	**96.43**

Specifically, it improves AUC by 4.0 percentage points over the best-performing baseline and increases AUPR by 7.1 percentage points. In addition, it achieves a 1.7-percentage point higher F1-score, while maintaining comparable recall. These results demonstrate better generalization under temporal distribution shifts and highlight the suitability of our model for real-world clinical deployment.

### Effect of similarity loss weight

We systematically analyze the balance between the binary cross-entropy loss (weight =1−η) and the feature similarity loss (weight =η) in the hybrid loss function to investigate the role of representation alignment between genomic and pseudo-genomic features during model training. The goal is to understand how the weighting of the similarity term affects feature discriminability and model generalization, rather than to perform hyperparameter optimization.

**Fig. 5.**
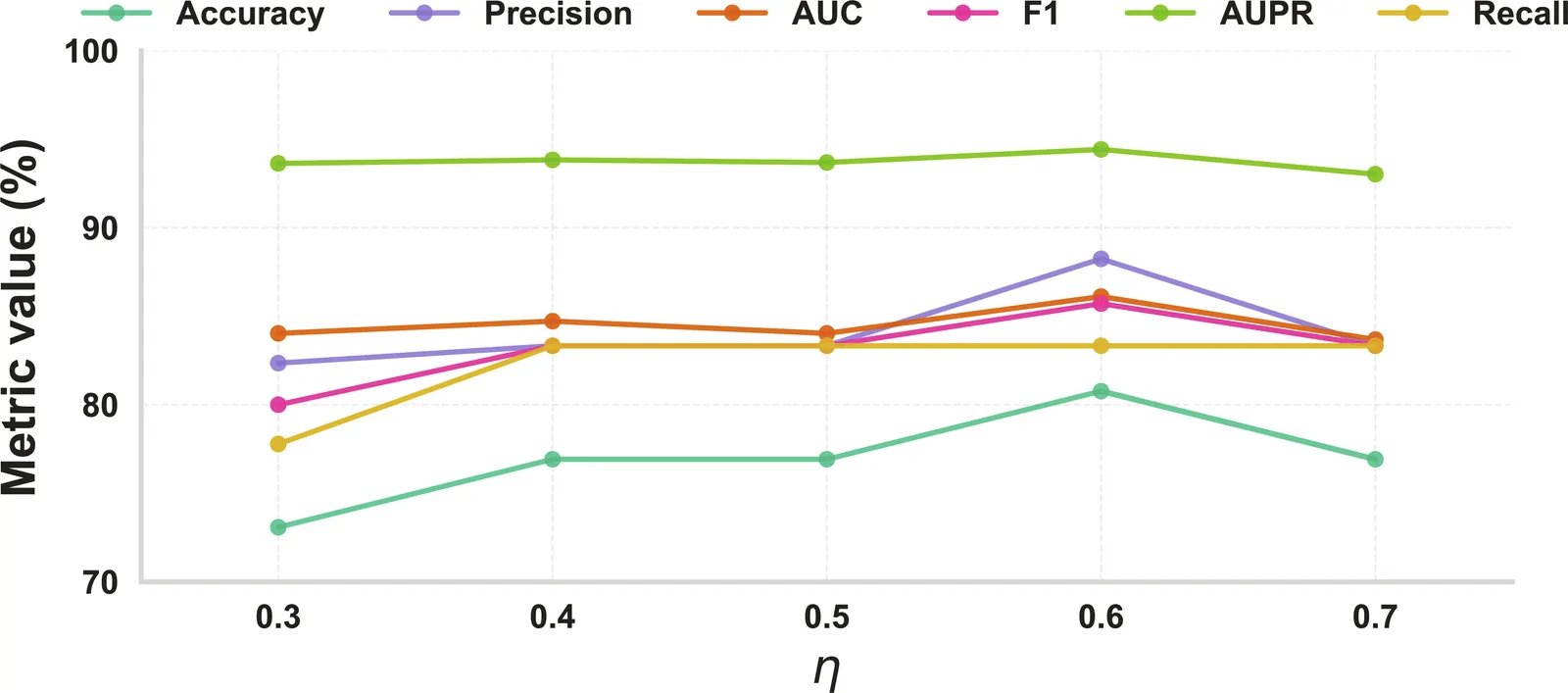
Hyperparameter experiments on loss weight η.

As shown in Fig. 5, model performance varied noticeably with different values of η, with relatively strong performance observed around η = 0.6. These results are based on a single train–test split. Under this setting, the model achieved competitive accuracy and AUC, slightly outperforming the configuration at η = 0.4 by 1.39%. These findings suggest that a moderate alignment constraint between genomic and pseudo-genomic representations can improve the discriminability and robustness of the learned feature space, thereby enhancing overall predictive performance. However, increasing η to 0.7 caused a substantial performance decline, with AUC decreasing by 2.43%, implying that excessive emphasis on representation alignment may over-constrain the latent space and weaken the model's focus on the downstream classification objective. Conversely, when η was reduced to 0.3, the similarity constraint became too weak to effectively guide the pseudo-genomic representation toward informative genomic structure, leading to reduced sensitivity to key molecular signatures.

Overall, these results indicate that η=0.6 provides a reasonable trade-off between supervised prediction and cross-modal representation alignment. In this work, we adopt this value based on the above analysis. Under this setting, the genomic modality can better guide multimodal representation learning, while the similarity objective improves robustness without overwhelming the classification target. These findings further highlight the importance of high-quality genomic representation in multimodal clinical drug response prediction.

### Explainability analysis

To interpret the contribution of gene features to model prediction, we applied SHAP (SHapley Additive exPlanations) analysis to the gene modality. Since the proposed model integrates gene features, PET-CT imaging reports, pathological and immunological indicators, and clinical information, we fixed the nongene modalities during SHAP computation and only perturbed the gene features. Therefore, the obtained SHAP explanations reflect the conditional contribution of gene features to the model prediction given the other modality information.

**Fig. 6.**
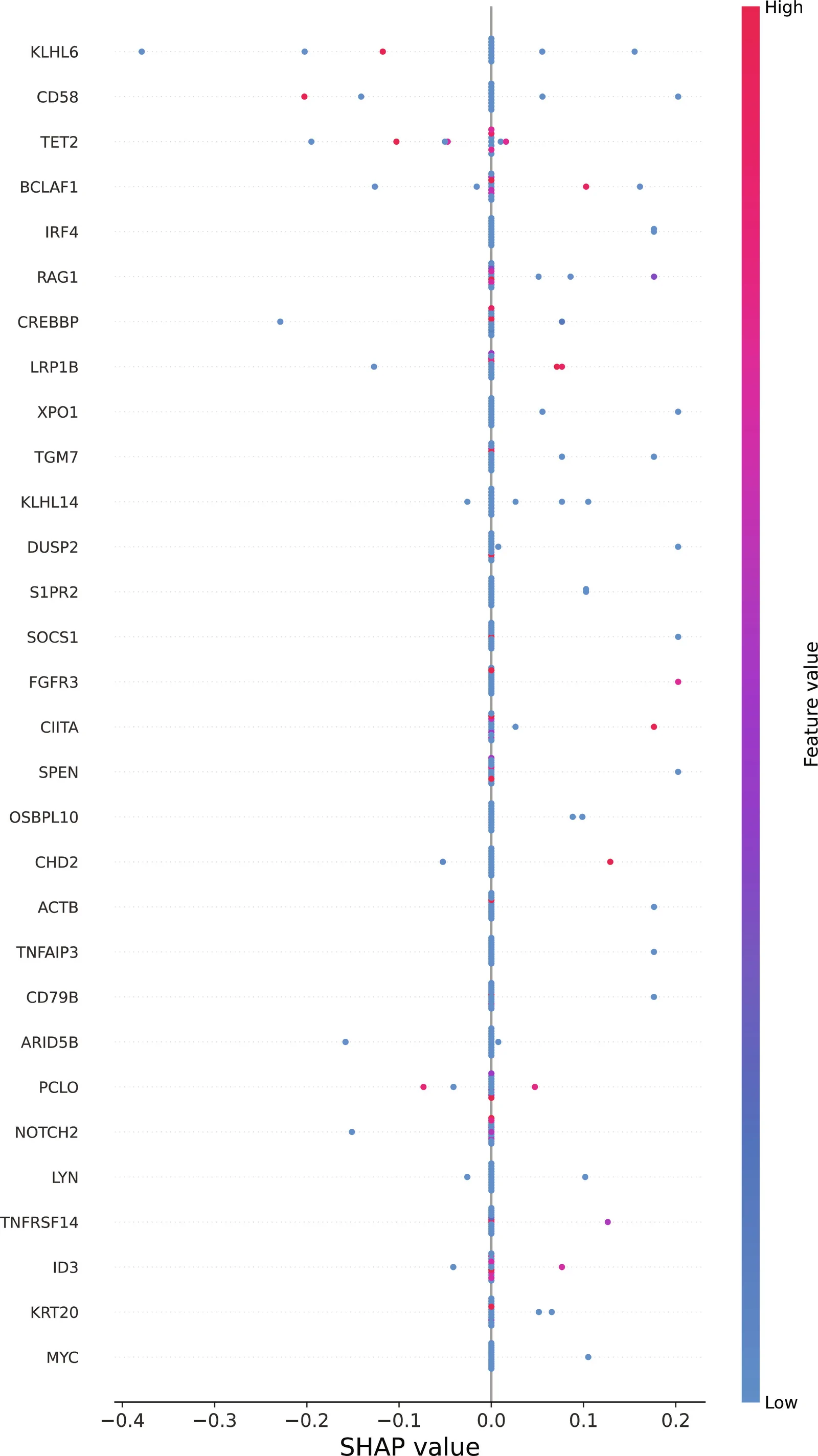
Feature importance based on SHAP values.

Based on this SHAP analysis, we further performed an interpretability assessment on a subset of samples from the independent test cohort, and the results are presented in Fig. 6. Mapping the top-ranked genomic features to biological pathways revealed that the model predominantly captures signals associated with B-cell receptor (BCR) signaling, transcriptional regulation, and epigenetic modification pathways. Key genes such as KLHL6, CD79B, and LYN are known components of the BCR signaling cascade, which governs antigen recognition and B-cell activation; notably, KLHL6 has been reported to play a critical role in germinal center formation and BCR-mediated responses [35,36].

In addition, transcription factors including IRF4, MYC, and CIITA constitute a central regulatory network controlling B-cell proliferation, differentiation [37–39], and class-switch recombination, consistent with their strong positive SHAP contributions observed in the selected test samples. Meanwhile, genes such as TET2, CREBBP, and ARID5B are involved in epigenetic regulation, including DNA methylation and chromatin remodeling [40,41]. Their predominantly negative SHAP values indicate that these features contributed negatively to the model output in the analyzed samples, suggesting potential modulatory effects within the predictive model.

Collectively, these findings suggest that the model may capture biologically meaningful and pathway-consistent genomic signals at the individual-sample level. However, this SHAP-based interpretation remains exploratory and should not be regarded as formal pathway-level biological validation.

## Conclusion

In this study, we proposed KeM-DRP, a knowledge-enhanced multimodal framework with genomic reconstruction for drug response prediction in DLBCL under realistic incomplete multimodal data conditions. Motivated by the heterogeneity of clinical multimodal data and the frequent unavailability of genomic sequencing in routine practice, we formulated drug response prediction as a multimodal learning problem with incomplete genomic information and validated the proposed framework on a real-world DLBCL cohort. By integrating biologically informed genomic representation learning, cross-modal genomic reconstruction, and genomics-guided adaptive fusion, KeM-DRP effectively incorporates genomic information into multimodal prediction while maintaining predictive capability when genomic data are missing or incomplete. Experimental results demonstrate that KeM-DRP consistently outperforms competitive baseline methods, highlighting the potential value of incorporating biological prior knowledge and adaptive multimodal integration for drug response prediction under incomplete data scenarios.

Nevertheless, this study has several limitations, including the relatively small cohort size and single-institutional setting. Although temporal split evaluation provides a more realistic assessment than random cross-validation, it remains an internal validation strategy and cannot fully capture potential variations across institutions. Future studies will focus on validating KeM-DRP in larger, independent, and multicenter cohorts to further assess its robustness and generalizability. In addition, improving model interpretability will be an important direction to better characterize multimodal feature contributions and facilitate a deeper understanding of the biological mechanisms underlying drug response prediction.

## Data Availability

The source code of KeM-DRP has been publicly released in the GitHub repository: https://github.com/bioinformatics-xu/KeM-DRP. The repository also provides example data, usage instructions, and the latest access information for the web-based platform.
