# Supplementary material for: A Knowledge-Enhanced Multimodal Framework with Genomic Reconstruction for DLBCL Drug Response Prediction
Source: Comput Struct Biotechnol J. 2026 Sep 2;35(1):0191. doi: 10.34133/csbj.0191 (PMC13534803; doi:10.34133/csbj.0191)
Supplement: Supplementary file 1 [file csbj.0191.f1.pdf]

Table S1: Gene features included in this study

Group	Gene List
A	ABCA13, ACTB, ACTRT3, ADAM21, ALPK3, AMOTL1, ANKRD11, ANKRD26, AP3B1, APC, APOB, ARID1A, ARID2, ARID5B, ASXL3, ATG2B, ATM, ATP7B, ATRX
B	B2M, BCL10, BCL11A, BCL2, BCL6, BCL7A, BCLAF1, BCOR, BIRC3, BMPR1A, BPTF, BRAF, BRCA1, BRCA2, BRIP1, BTG1, BTG2, BTK
C	CACNA1H, CACNA1S, CAD, CARD11, CCDC129, CCND1, CCND2, CCND3, CD36, CD58, CD70, CD79A, CD79B, CD93, CDKN1A, CDKN1B, CHD2, CHD8, CHEK2, CHMP1A, CHPF, CHST2, CIC, CIITA, CLGN, COL12A1, CREBBP, CROCC, CRYBG1, CSMD1, CTLA4, CTNNA3, CUX1, CXCR1, CXCR4, CXCR5
D	DAPK1, DDX11, DDX3X, DHX15, DIS3, DNAH10, DOT1L, DSC2, DSG2, DSP, DTX1, DUSP2
E	EBF1, ECSIT, ECT2L, EFL1, EGFR, EGR2, EIF2A, EOMES, EP300, EPHA1, EPHA10, ERCC6L2, ERG, ETS1, ETV1, ETV6, EZH2
F	FAS, FAT1, FAT3, FAT4, FBN1, FBXW7, FGFR3, FLT3, FOXC1, FOXF1, FOXO1, FOXO3
G	GATA2, GNA13, GNLY, GRHPR, GZMB
H	HASPIN, HDAC9, HIST1H1C, HIST1H1D, HIST1H1E, HIST1H2BK, HIST2H2AB, HLA-A, HLA-B
I	ID3, IGLL5, IKBKB, IKZF1, IKZF3, IL16, IL2RB, IL7R, INPP5B, IRF4, IRF8, ITPKB, ITPR3
J	JAK2, JMY
K	KDM5A, KDM6A, KDM6B, KDR, KIR2DL1, KIT, KLF2, KLHDC4, KLHL14, KLHL21, KLHL6, KMT2A, KMT2B, KMT2C, KMT2D, KRAS, KRT20, KRT6C
L	LAMA3, LAMP1, LRP1B, LRRN3, LYN
M	MALT1, MAP3K5, MBD4, MECOM, MED12, MEF2B, MEN1, MGA, MKI67, MPEG1, MSH3, MSH6, MTOR, MUC16, MUC4, MUTYH, MYC, MYD88, MYH11, MYOM2
N	NAF1, NBEA, NCOR1, NCOR2, NFATC1, NFE2, NFKBIE, NLE1, NOL9, NOS2, NOTCH1, NOTCH2, NOTCH3, NRAS, NRCAM, NRXN3, NSD1, NSD2, NT5C2
O	OSBPL10
P	PAX5, PCLO, PCSK9, PDCD1, PDCD11, PDGFRA, PEG3, PHIP, PIK3CA, PIK3CD, PIK3CG, PIK3R1, PIK3R2, PIM1, PIM2, PLCG1, PLCG2, PLXNB3, PML, PMS1, PMS2, POF1B, POSTN, POT1, POU2F2, PRDM1, PRPF40B, PTEN, PTPN13, PTPN6, PTPRC, PTPRD, PTPRK, PTPRQ, PTPRT, P2RY8
R	RAD21, RAG1, RAX, RECQL4, RET, REV3L, RHPN2, ROBO2, ROS1, RP1L1, RPAP1, RPE, RPS15, RRAGC, RTEL1, RYR2
S	S1PR2, SALL3, SAMD9, SAMD9L, SAMHD1, SCAF1, SCG3, SCN5A, SELPLG, SETBP1, SETD1B, SETD2, SGK1, SH2B3, SIN3A, SLITRK3, SMARCA2, SOCS1, SP140, SPEN, SPI1, SRGN, STAG1, STAG2, STAT3, STAT5B, STAT6, STXBP2
T	TBL1XR1, TCF3, TERT, TET1, TET2, TGM7, TIAM1, TMEM30A, TMEM43, TMSB4X, TNFAIP3, TNFRSF10A, TNFRSF10B, TNFRSF14, TOX, TP53, TRAF2, TRAF3, TRAM1L1, TRIP12, TRRAP, TSC1, TSC2, TTN, TYK2
U	UBE2A, UNC13D, USP45, USP6
V	VPREB1, VPS13B
W	WDR90, WEE1
X	XPO1
Y	YEATS2
Z	ZEB1, ZFP36L1, ZMYM3, ZNF608, ZNF90
